# Chromosomal instability in circulating tumor cells and cabazitaxel resistance in metastatic castration-resistant prostate cancer

**DOI:** 10.1172/jci.insight.196505

**Published:** 2025-11-04

**Authors:** Ossian Longoria, Jan Rekowski, Santosh Gupta, Nick Beije, Klaus Pantel, Eleni Efstathiou, Cora Sternberg, Daniel Castellano, Karim Fizazi, Bertrand Tombal, Adam Sharp, Oliver Sartor, Sandrine Macé, Christine Geffriaud-Ricouard, Richard Wenstrup, Ronald de Wit, Johann de Bono

**Affiliations:** 1 The Institute of Cancer Research, London, United Kingdom.; 2 The Royal Marsden NHS Foundation Trust, London, United Kingdom.; 3 Clinical Trials and Statistics Unit at The Institute of Cancer Research, London, United Kingdom.; 4 Epic Sciences, San Diego, California, USA.; 5 Erasmus MC Cancer Institute, Rotterdam, Netherlands.; 6 University Medical Center Hamburg-Eppendorf, Hamburg, Germany.; 7 Houston Methodist Cancer Center, Houston, Texas, USA.; 8 Englander Institute for Precision Medicine, Weill Cornell Medicine, Meyer Cancer Center, New York, New York, USA.; 9 Hospital Universitario 12 de Octubre, Madrid, Spain.; 10 Institut Gustave Roussy, University of Paris-Saclay, Villejuif, France.; 11 Université Catholique de Louvain, Cliniques Universitaires Saint-Luc, Brussels, Belgium.; 12 Mayo Clinic Comprehensive Cancer Center, Rochester, Minnesota, USA.; 13 Sanofi, Paris, France.

**Keywords:** Clinical Research, Oncology, Biomarkers, Clinical trials, Prostate cancer

## Abstract

**BACKGROUND:**

Predictive biomarkers to guide chemotherapy decisions for metastatic castration–resistant prostate cancer (mCRPC) are lacking. Preclinical studies indicate that circulating tumor cell (CTC) studies of chromosomal instability (CTC-CIN) can predict taxane resistance.

**METHODS:**

The CARD trial randomized individuals with mCRPC progressing within a year of treatment with an androgen receptor pathway inhibitor (ARPI; enzalutamide or abiraterone acetate plus prednisolone/prednisone) to cabazitaxel or the alternative ARPI. As a preplanned biomarker analysis, CTCs were isolated from blood samples obtained at baseline, cycle 2, and the end of treatment. Associations between baseline CTC and CTC-CIN counts with imaging-based progression-free survival (ibPFS), overall survival (OS), time to prostate-specific antigen (PSA) progression, RECIST 1.1 objective response rate (ORR), and PSA_50_ response rate were assessed.

**RESULTS:**

High baseline CTC-CIN counts significantly associated with worse OS after adjustment for confounding variables (median OS, 15.3 vs. 8.9 months; univariate HR, 2.16; 95% CI, 1.52–3.06; *P* < 0.001; multivariate HR, 1.56; 95% CI, 1.01–2.43; *P* = 0.047). Detectable CTC-CIN counts at baseline may predict a lack of ibPFS and OS benefit when comparing cabazitaxel with ARPI.

**CONCLUSION:**

This preplanned analysis of biomarker data from the CARD trial confirms that CTC-CIN counts are a clinically useful prognostic and predictive biomarker of taxane resistance in mCRPC. Detectable CTC-CIN at baseline defines a patient subpopulation with unmet clinical needs in which alternative therapeutics should be tested.

**TRIAL REGISTRATION:**

ClinicalTrials.gov number NCT02485691.

**FUNDING:**

Funded by Sanofi and Epic Sciences.

## Abstract 2

In this sub-analysis of a landmark clinical trial, circulating tumor cells with features of chromosomal instability predicts resistance to chemotherapy in prostate cancer patients.

## Introduction

The randomized CARD trial demonstrated that cabazitaxel significantly improved imaging-based progression-free survival (ibPFS) and overall survival (OS) versus abiraterone or enzalutamide in patients with metastatic castration–resistant prostate cancer (mCRPC) previously treated with docetaxel and who had progressed within 12 months on the alternative androgen receptor pathway inhibitor (ARPI), thus establishing cabazitaxel as the standard of care for heavily pretreated patients with mCRPC (1). Despite this, survival gains over a second ARPI are limited, and toxicity, especially febrile neutropenia, remains a significant concern. Although dose reduction of cabazitaxel from 25 mg/m^2^ to 20 mg/m^2^ produces a noninferior survival outcome, with an improved safety profile and less cost, as granulocyte colony growth factor (G-CSF) may be avoided, response rates significantly decrease with reduced dosing, which suggests that some patient subgroups, such as patients with aggressive prostate cancer variants harboring combined pathogenic alterations of *PTEN*, *RB1*, or *TP53* or patients with tumor-induced neutrophilia, may yet benefit from a higher dose intensity (2–4). Novel treatments like Lu^177^ vipivotide tetraxetan have not demonstrated superior survival outcomes over cabazitaxel but have proved to be better tolerated (5). To date, the only validated biomarkers predicting treatment benefit for patients with mCRPC are the presence of DNA repair defects, such as *BRCA1* or *BRCA2* pathogenic alterations, which can provide information on susceptibility to PARP1/2 inhibitors; PSMA expression, as estimated by PSMA-PET SUV_mean_, which predicts susceptibility to Lu^177^ vipivotide tetraxetan; and PSMA-PET total tumor volume, which predicts clinical benefit for the combination of Lu^177^ vipivotide tetraxetan and enzalutamide in first-line mCRPC (6–9). Predictive biomarkers that improve patient selection for life-prolonging but potentially toxic drugs like taxanes are urgently needed.

Circulating tumor cell (CTC) counts, utilizing enrichment methods, including EpCAM-based capture, are a validated pretreatment prognostic measure with regulatory clearance for routine clinical use in patients with metastatic castration-resistant prostate cancer (mCRPC) (10–15). The non-enrichment-based EPIC Sciences CTC platform evaluates cytokeratin^+^ (CK^+^) cells in circulation for morphologic, molecular, and genomic characterization of CTCs (16–21). This integrated analysis can inform on CTC counts, AR-V7 splice variant presence, and neuroendocrine morphology as well as indirectly estimate chromosomal instability (CIN) through morphological prediction of large-scale transitions (LSTs), which are chromosomal gains or losses greater than 10 megabases. CIN, as estimated by this method, has been associated with worse survival after ARPI and taxane treatment in clinical validation studies and is a hallmark of cancer directly linked to disease aggressiveness and drug resistance across tumor types (20–23). Pan-cancer genomic analyses of CIN indicate that metastatic prostate cancer is enriched for high CIN and is the tumor type with the strongest correlation between CIN and metastatic burden, especially liver and bone metastases (24, 25). CIN has been reported to associate with taxane resistance preclinically, but its interrogation has not been rigorously pursued in clinical trials (26, 27).

A preplanned biomarker analysis of the CARD study investigated CTC counts, defined as any circulating CK^+^, cluster of differentiation 45^–^ (CD45^–^) intact cell on the EPIC Sciences CTC platform, as a biomarker of prognosis and response to therapy in patients receiving either cabazitaxel or an ARPI. We hypothesized that a morphology-informed assessment of CIN in CTCs would predict clinical outcomes with cabazitaxel in mCRPC.

## Results

### Patient population

Overall, 255 patients with mCRPC were randomized to receive cabazitaxel (*n* = 129) or a second ARPI (*n* = 126). Median follow-up was 9.2 months and the median duration of treatment with cabazitaxel was 22 weeks (range: 13.1–30.4 weeks) compared with 12.5 weeks (range: 9.9–23.4 weeks) with a second ARPI. CTC counts were obtained from 237 patients at screening, 213 patients at cycle 2, day 1 (C2D1) and 166 patients at the end of treatment (EOT) (Supplemental Figure 1; supplemental material available online with this article; https://doi.org/10.1172/jci.insight.196505DS1). Thirty-five samples (5.1%) failed quality control (QC) criteria. CTC and CTC-CIN count availability and distribution per time point are summarized in Supplemental Table 1 and Supplemental Figure 2. One or more CTCs were detected in 204 samples at baseline (86%), in 178 samples at C2D1, and in 132 samples at EOT. Median (minimum to maximum) CTC and CTC-CIN counts at each time point are summarized in Supplemental Table 2. Overall, 49.37% of individuals had low CTC counts at baseline and 50.63% had high baseline CTC counts. Baseline characteristics for the patients with evaluable CTC and CTC-CIN samples are summarized in Supplemental Tables 3 and 4, respectively. Patients with high CTC counts at baseline had higher prostate-specific antigen (PSA), lactate dehydrogenase (LDH), alkaline phosphatase (ALP), and pain or analgesic use at baseline; patients with high CTC-CIN were also more likely to have visceral metastases.

### Association between baseline CTC-CIN counts and time-to-event outcomes

#### OS.

**Figure 1:**
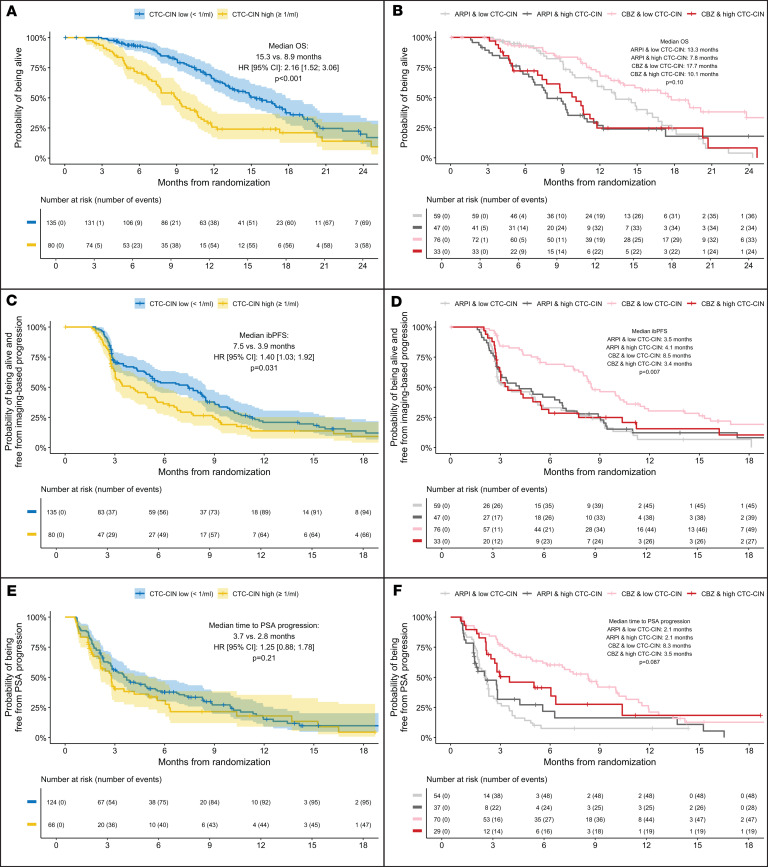
Kaplan-Meier curves of OS, ibPFS, and TTPP from randomization by baseline CTC-CIN count irrespective of treatment arm and by treatment arm. (**A**). OS irrespective of treatment arm. (**B**) OS by treatment arm. (**C**) ibPFS irrespective of treatment arm. (**D**) ibPFS by treatment arm. (**E**) TTPP irrespective of treatment arm. (**F**) TTPP by treatment arm.

High baseline CTC-CIN counts were significantly associated with worse survival (median OS, 15.3 vs. 8.9 months; univariate HR, 2.16; 95% CI 1.52–3.06; *P* < 0.001; Figure 1A); multivariate analysis confirmed this finding (HR, 1.56; 95% CI, 1.01–2.43; *P* = 0.047; Table 1). The variables significantly associated with OS in this model were treatment arm; log_10_-transformed PSA, LDH, and neutrophil-lymphocyte ratio (NLR); Eastern Cooperative Oncology Group (ECOG) performance status of 2; presence of visceral disease at baseline; and Gleason score of 8 or greater at diagnosis. An accelerated failure time (AFT) Weibull model confirmed that OS depended on baseline CTC-CIN count, whether analyzed irrespective of χ^2^_2_ = 25.3, *P* < 0.001) or adjusted for treatment arm (χ^2^_2_ = 21.1, *P* < 0.001; Supplemental Figure 3, A and B). Analysis by treatment arm suggested no difference in risk of death between treatment arms if CTC-CIN was high at baseline; however, a statistically significant interaction between CTC-CIN count and treatment arm was not found (ARPI and CTC-CIN low, 13.3 months; cabazitaxel and CTC-CIN low, 17.7 months; ARPI and CTC-CIN high, 7.8 months; cabazitaxel and CTC-CIN high, 10.1 months; *P* for interaction = 0.1; Figure 1B).

#### ibPFS.

Irrespective of treatment arm, high CTC-CIN at baseline associated with worse ibPFS (median ibPFS, 7.5 months vs. 3.9 months; HR, 1.4; 95% CI, 1.03–1.92; *P* = 0.031; Figure 1C). Analysis by treatment arm indicated that having a low CTC-CIN count at baseline predicted a statistically and clinically significant gain in ibPFS from cabazitaxel and, conversely, that having a high baseline CTC-CIN count predicted no difference in ibPFS regardless of treatment (3.5 months for ARPI and CTC-CIN low, 4.1 for ARPI and CTC-CIN high, 8.5 for cabazitaxel and CTC-CIN low, and 3.4 for cabazitaxel and CTC-CIN high; *P* for interaction = 0.007; Figure 1D). An AFT Weibull model suggested that ibPFS depended on CTC-CIN count when analyzed irrespective of χ^2^_2_ = 7, *P* < 0.03) but not when adjusted for treatment arm (χ^2^_2_ = 2.4, *P* = 0.295; Supplemental Figure 3, C and D).

#### Time to PSA progression.

Overall, higher CTC-CIN counts at baseline were not associated with worse time to PSA progression (TTPP) by either univariate or multivariate analyses or AFT Weibull models (Figure 1E, Table 1, and Supplemental Figure 3E). When analyzed by treatment arm, however, TTPP was considerably longer for patients with low baseline CTC-CIN treated with cabazitaxel, although a statistically significant interaction between CTC-CIN levels and treatment arm was not demonstrated (2.1 months for ARPI and CTC-CIN low, 2.1 for ARPI and CTC-CIN high, 8.3 for cabazitaxel and CTC-CIN low, and 3.5 for cabazitaxel and CTC-CIN high; *P* for interaction = 0.087; Figure 1F and Supplemental Figure 3F).

### Association between baseline CTC counts and time-to-event outcomes

#### OS.

**Figure 2:**
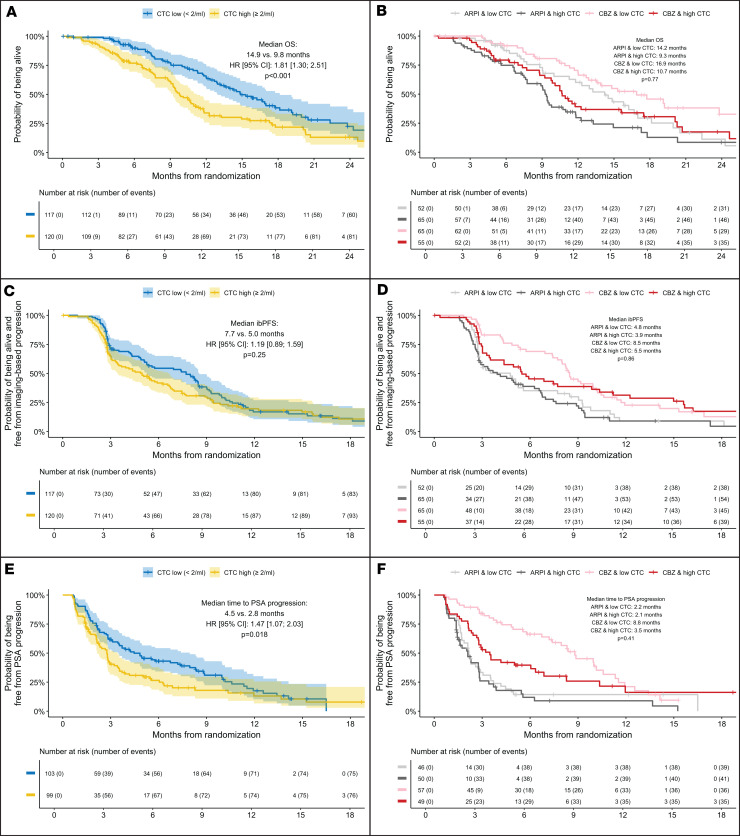
Kaplan-Meier curves of OS, ibPFS, and TTPP from randomization by baseline CTC count irrespective of treatment arm and by treatment arm. (**A**) OS irrespective of treatment arm. (**B**) OS by treatment arm. (**C**) ibPFS irrespective of treatment arm. (**D**) ibPFS by treatment arm. (**E**) TTPP irrespective of treatment arm. (**F**) TTPP by treatment arm.

High baseline CTC counts were significantly associated with worse OS in the overall population in univariate analysis (median OS, 9.8 vs. 14.9 months; univariate HR, 1.81; 95% CI, 1.30–2.51; *P* < 0.001; Figure 2A). Kaplan-Meier analysis did not show an interaction between treatment arm and CTC counts at baseline (*P* for interaction = 0.77; Figure 2B). In multivariable analysis, the HR for high versus low CTC count was 1.43 (95% CI, 0.95–2.13), although this was not statistically significant (Supplemental Table 5). An AFT Weibull model indicated that OS depended on baseline CTC count, whether analyzed irrespective of χ^2^_2_ = 7, *P* = 0.03) or adjusted for treatment arm (χ^2^_2_ = 7.6, *P* = 0.023; Supplemental Figure 4, A and B).

#### ibPFS.

Low baseline CTC counts were not significantly associated with prolonged ibPFS (7.7 vs. 5.0 months; univariate HR, 1.19; 95% CI, 0.89–1.59; *P* = 0.25; Figure 2C). Multivariate and AFT Weibull model analysis reflected this finding (Supplemental Table 5 and Supplemental Figure 4, C and D). There was no indication of an interaction between baseline CTC count and treatment arm (*P* for interaction = 0.86, Figure 2D).

#### TTPP.

Low baseline CTC counts were significantly associated with prolonged TTPP in the overall population (median TTPP, 4.5 vs. 2.8 months; univariate HR, 1.47; 95% CI, 1.07–2.03; *P* = 0.018; Figure 2E). Multivariate Cox regression analysis and a parametric AFT Weibull model were not consistent with this observation (Supplemental Table 5 and Supplemental Figure 4, E and F, respectively). There was no indication of an interaction between baseline CTC count and treatment arm (*P* for interaction = 0.41; Figure 2F).

### Association between baseline CTC and CTC-CIN counts and tumor response

#### Radiological response.

**Figure 3:**
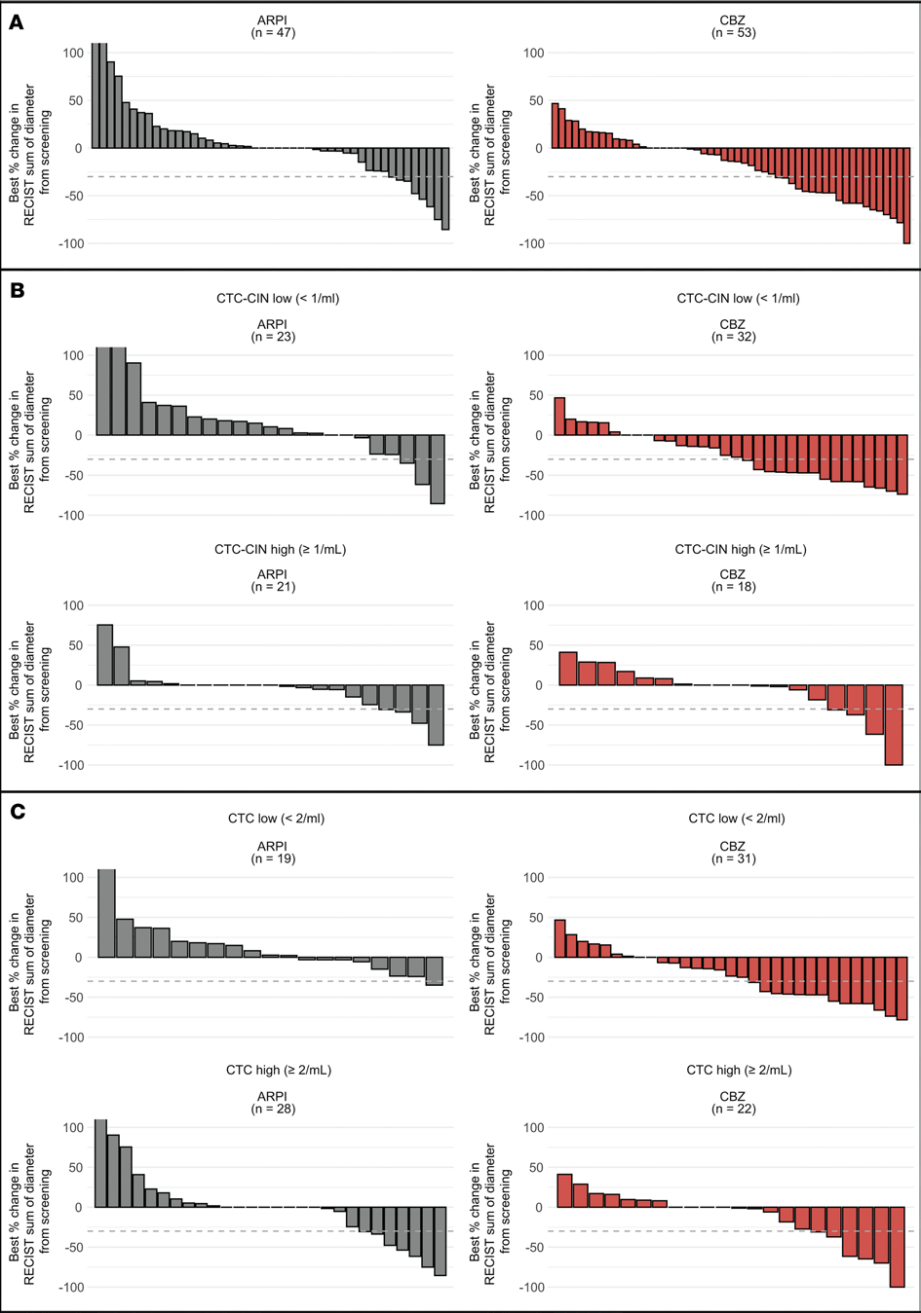
Best RECIST 1.1 response waterfall plots. (**A**) Best RECIST 1.1 response (BRR) by treatment arm. (**B**) BRR by baseline CTC count. (**C**) BRR by baseline CTC-CIN count.

ORRs by CTC counts at baseline, when analyzed by treatment group, indicated a greater magnitude of benefit with cabazitaxel over ARPI across both CTC and CTC-CIN high/low and present/absent groups (Figure 3 and Supplemental Table 6). CTC-CIN counts at baseline predicted cabazitaxel resistance; overall, 16% of patients with low CTC-CIN counts at baseline and 54% of patients with high CTC-CIN counts at baseline had progressive disease as their best overall response (BOR; OR for progressive disease if CTC-CIN low at baseline, 0.17; *P* = 0.003; Supplemental Table 7). CTC-CIN did not discriminate BOR among patients treated with ARPI, and CTC counts were not accurate discriminators of BOR. Of 190 patients with measurable disease, 27 had a partial response; no complete responses were observed; 19 had a duration of response of 24 weeks or longer. Of these, 13 were CTC-CIN low at baseline and received cabazitaxel, 2 were CTC-CIN high and received cabazitaxel, 3 were CTC-CIN high and received ARPI, and 1 patient randomized to cabazitaxel was not evaluable for CTC-CIN (Supplemental Figure 5).

#### Biochemical response.

**Figure 4:**
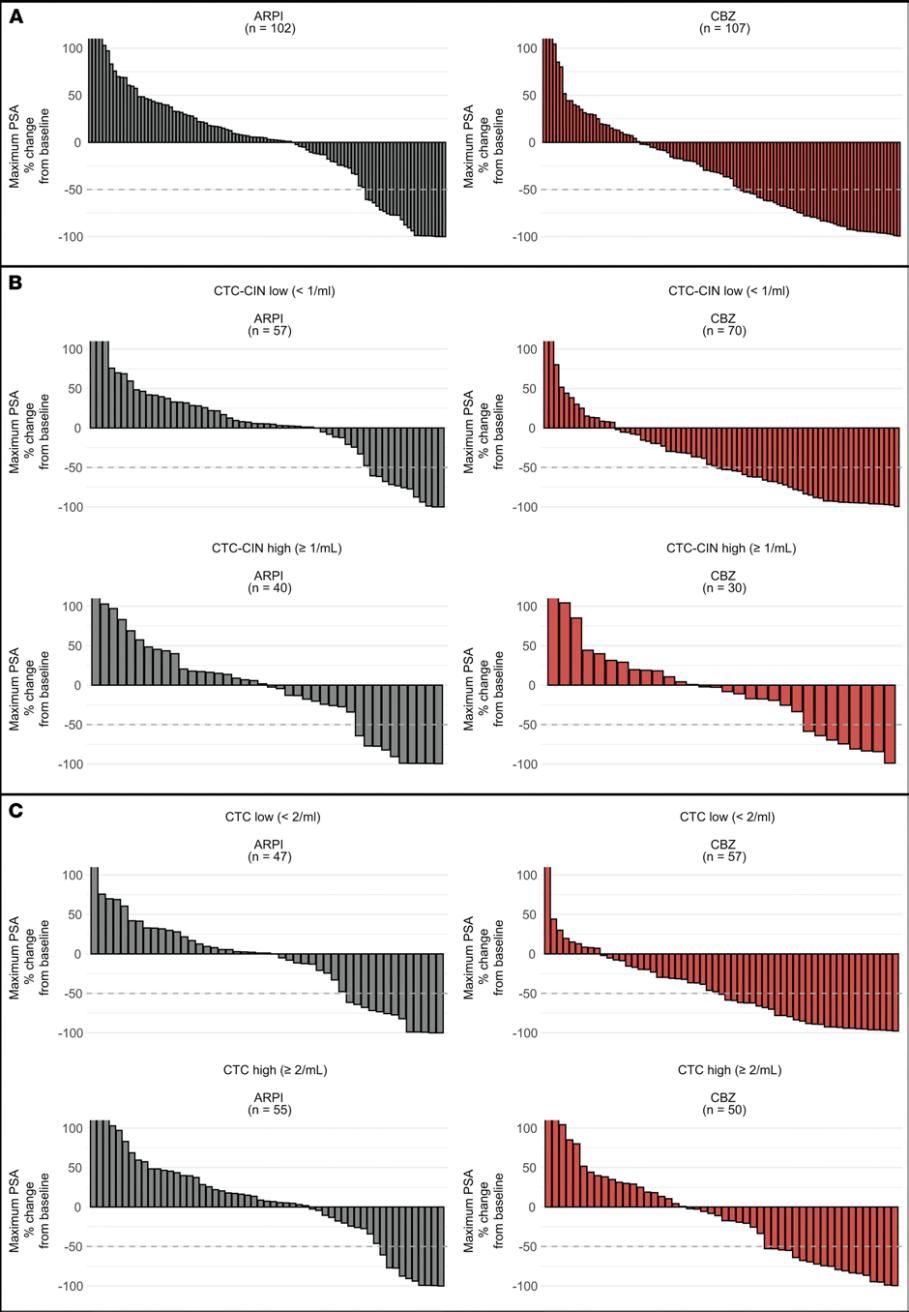
Best PSA response (BPR) waterfall plots. (**A**) Best PSA response (BPR) by treatment arm. (**B**) BPR by baseline CTC count. (**C**) BPR by baseline CTC-CIN count.

PSA_50_ response rates (PRR; PSA_50_ is defined as a 50% or greater reduction in PSA from baseline confirmed at least a week later) followed a similar trend to the above ORR data when analyzed by treatment groups, with a greater magnitude of benefit observed with cabazitaxel over ARPI across both CTC and CTC-CIN high/low and present/absent groups (Figure 4 and Supplemental Table 8). CTC-CIN counts at baseline were a good discriminator of PRR for patients randomized to cabazitaxel (CTC-CIN low, 41%; CTC-CIN high, 20%) but not ARPI (CTC-CIN low, 14%; CTC-CIN high, 13%). Overall, of 209 evaluable patients, 53 had a PSA_50_ response. Patients with low baseline CTC-CIN counts had significantly lower odds of not achieving a PSA_50_ response if randomized to cabazitaxel (OR 0.36; *P* = 0.04; Supplemental Table 9). Twenty-eight patients had a duration of PSA_50_ response of 24 weeks or longer; of these, 17 patients were CTC-CIN low at baseline and received cabazitaxel, 4 were CTC-CIN high and received cabazitaxel, 3 were CTC-CIN low and received ARPI, 2 were CTC-CIN high and received ARPI, and 2 patients were not evaluable for CTC-CIN; and 1 was randomized to ARPI and 1 to cabazitaxel (Supplemental Figure 6).

### Interaction between baseline CTC and CTC-CIN counts and tumor response

Since CTC-CIN is a less frequent circulating cell type than CTC, high CTC-CIN at baseline may be a better prognostic measure than CTC alone because it represents a patient subpopulation with highest CTC counts. Including both CTC and CTC-CIN counts in multivariable regression models may be problematic due to perfect collinearity, as CTC-CINs are a subset of CTCs. To gain a better understanding of the relationship between CTC and CTC-CIN baseline counts and clinical outcomes, we plotted these 2 groups against each other (Supplemental Figure 7). Among individuals with low baseline CTC-CIN counts, those with low baseline CIN counts had a median OS of 15.8 months and those with high CTC counts at baseline a median OS of 14.9. Conversely, among individuals with high CTC-CI counts, those with low baseline CTC counts had a median OS of 6.3 months and those with high CTC counts at baseline had a median OS of 9.2 months (*P* for interaction = 1).

## Discussion

The results of this preplanned analysis of CTC analyses with a nonenrichment assay for patients on the prospective randomized CARD trial indicate that baseline nonenriched CTC counts correlate with OS but not with ibPFS, TTPP, or ORR. This is consistent with the findings of previous studies for phase III mCRPC trials with enrichment CTC assays (10–15). Although extensively validated in clinical trials, CTC enumeration has not been widely adopted in routine clinical care because it rarely informs treatment decisions (28). In this study, cabazitaxel outperforms a second ARPI regardless of baseline CTC counts, which underscores the findings of the CARD trial: patients with mCRPC progressing within a year of starting an ARPI have a poor prognosis and do not benefit from a second ARPI (1).

A high CTC-CIN count at baseline was also associated with worse OS irrespective of treatment arms, but patients with high CTC-CIN randomized to cabazitaxel (80 of 215 assessable patients, 37.21%) showed poor outcomes comparable to patients treated with ARPI. ibPFS, the primary endpoint of CARD, was no different in patients with CTC-CIN at baseline treated with cabazitaxel (3.4 months) and patients treated with an ARPI, regardless of baseline CTC-CIN count (low CTC-CIN: 3.5 months, high CTC-CIN: 4.1 months). Likewise, the TTPP and OS curves for CTC-CIN patients overlapped, suggesting no benefit of cabazitaxel over ARPI in men with high baseline CTC-CIN. This is concordant with prior validation studies of this assay and clinically confirms preclinical observations that CIN is linked to taxane resistance (20). A high CIN gene signature has been previously associated with paclitaxel resistance and carboplatin sensitivity in the OV01 ovarian cancer clinical trial (26). More recently, pan-cancer CIN signatures derived from copy number profiles from The Cancer Genome Atlas were found to associate with taxane resistance in retrospectively emulated biomarker clinical trials using real-world data from patients with ovarian, breast, and prostate cancer (29).

This nonenriched CTC assay identifies CTC-CIN by predicting the presence of ≥9 LSTs per CTC by morphology alone (20). CIN is also associated with other genomic features like alterations in tumor suppressor genes (TSGs) such as *PTEN*, *RB1*, and *TP53* (25). Aggressive-variant prostate cancer (AVPC), as the name suggests, is a clinically aggressive subtype of non–small cell prostate cancer enriched for combined alterations in *PTEN*, *RB1*, or *TP53* (30). Corn et al. randomized 160 patients with mCRPC to cabazitaxel (25 mg/m^2^) or cabazitaxel plus carboplatin (AUC 4). Combination treatment decreased the risk of PFS but not OS in the overall population (3). Patients with combined defects in *PTEN*, *RB1*, or *TP53* had improved PFS (HR 0.35, *P* < 0.001) and OS (HR 0.39, *P* = 0.002) when carboplatin was added to cabazitaxel. CTC analyses from this trial, performed with the Epic CTC assay, showed that single CTCs from patients with combined TSG defects had higher LST scores than patients without combined TSG alterations (median LST in CTC 28.7 vs. 9.5, *P* = 0.002) (31). Therefore, AVPC and CTC-CIN may represent two sides of the same coin, and CTC-CIN detection may help define a trial population to test treatment intensification strategies, novel drugs, and validate putative synthetic lethality targets for CIN (24, 27, 32).

Clinical utility drives the adoption of biomarkers. Somatic tumor testing is uniformly recommended for men with mCRPC because olaparib has been shown to prolong survival over abiraterone or enzalutamide in men with somatic or germline *BRCA1*, *BRCA2*, or *ATM* pathogenic alterations in the PROfound phase III trial (33–38). If CIN is a driver of taxane resistance, in principle it could be detected in tissue, ctDNA, or CTCs, but challenges remain. CIN can be estimated from cell-free DNA, but DNA tumor fractions higher than 5%–20% are required to accurately assess CNV changes (29, 39, 40). LST scores assessed through low-pass whole-genome sequencing of cfDNA from men treated in the PROSELICA and FIRSTANA trials were found to be prognostic of poor OS but could not discriminate between taxane responders and nonresponders (41). Although tissue next-generation sequencing remains the gold standard for accurate assessment of CIN, most samples tested in clinical practice are archival samples obtained at the time of diagnosis from the prostate primary (42, 43). Phylogenetic analyses suggest that most CNVs occur after metastasis, but measures of CIN such as LST scores are consistent across metastatic sites, despite differing baseline ploidies (32). Finally, a direct comparison of CIN measures in mechanistic models of CIN suggested that quantification of CIN in single cells through methods like cell imaging, cytogenetics, or single-cell DNA sequencing is superior to bulk genomic DNA or transcriptomic signatures (44). In summary, CTC-CIN has unique advantages when examining CIN to gain information about taxane resistance in men with mCRPC and difficult-to-biopsy sites of metastatic disease.

There are several limitations that should be considered when interpreting the results herein. The CARD study was powered to assess whether cabazitaxel was superior to abiraterone or enzalutamide in a specific setting and not to assess these biomarkers. Moreover, no orthogonal method to confirm CIN in CTCs was used in this study. However, the results with this nonenrichment-based CTC platform are consistent with prior validation (19–21).

In conclusion, this preplanned biomarker analysis of the prospective, randomized, CARD trial confirms that detection of CIN in CTCs from a single blood sample is feasible, that it is a more powerful prognosticator of OS than nonmorphology informed CTC count, and that it predicts taxane benefit or lack thereof in patients with mCRPC. Further studies incorporating this predictive assay to improve the standard of care arguably by treatment intensification or other therapeutic strategies in the population with positive CTC-CIN at baseline are now indicated.

## Methods

### Sex as a biological variable.

Prostate cancer afflicts males, therefore only males were considered for inclusion in this trial.

### Study design and patients.

CARD was a randomized, open-label clinical trial conducted at 62 sites across 13 European countries. Overall, 255 patients with mCRPC who had disease progression within 12 months of starting treatment with an ARPI (abiraterone acetate or enzalutamide) were randomly assigned in a 1:1 ratio to either cabazitaxel (25 mg/m^2^ every 3 weeks plus prednisolone/prednisone 10 mg daily and primary G-CSF prophylaxis) or a second ARPI (abiraterone acetate 1,000 mg once daily plus prednisolone/prednisone 5 mg twice daily or enzalutamide 160 mg once daily, whichever the patient had not received before) until confirmed radiographic progression, unacceptable toxicity, or refusal of further treatment. Full details of this trial have been previously reported (1). The primary endpoint was ibPFS from randomization as defined by RECIST 1.1 for measurable disease progression and by Prostate Cancer Working Group 2 (PCWG2) criteria for bone disease progression as determined locally by the investigator (45). Secondary outcomes included OS, TTPP, ORR (defined by RECIST 1.1 criteria in patients with measurable disease), and PSA_50_ response rate (PRR; defined as a 50% or greater reduction in PSA confirmed at least a week later). OS was defined as the time between the date of randomization and the date of death from any cause. TTPP was calculated as the time from randomization to first documented PSA progression per PCWG2 criteria. All CTC analyses, including CTC-CIN, were predefined, exploratory biomarker analyses of CARD.

### Sample collection and processing.

A single blood sample was collected in a Streck tube at screening (baseline); C2D1; and EOT visits and sent to Epic Sciences for CTC analysis. Samples were analyzed as described previously utilizing the Epic Sciences platform, a second-generation CTC detection technology that does not require enrichment or affinity isolation of rare cells (19). From a single tube of blood, nucleated cells were plated onto glass pathology slides, stained for pan-CK, CD45, the N-terminus of the androgen receptor (AR), and DAPI and analyzed by immunofluorescence and high-throughput imaging. CTCs were detected in silico by a proprietary machine vision algorithm and defined as cells with an intact nucleus that were CK^+^, CD45^–^, AR^+^ or AR^–^, and DAPI^+^; clusters of CTCs, defined as 2 or more adjoining cells, were counted as 1 event in the total count. Morphology of CTC subtypes determined the presence of CTC-CIN, as previously validated (20). The results were reported per mL of blood. Samples were excluded if they failed QC, including sample arrival after 96 hours or clotted.

### Statistics.

This CARD biomarker analysis assessed the association of CTC counts at baseline (dichotomized values per median; <2/mL cutoff) and CTC-CIN counts at baseline (dichotomized values per median; <1/mL cutoff) with ibPFS, OS, and ORR in the overall evaluable population; this was defined as randomized patients who had at least 1 evaluable sample. Samples were considered nonevaluable if they failed QC; were obtained at a time point other than baseline, C2D1, or EOT; or more than one sample existed per time point. All data analyses were performed using the R programming language version 4.3.1 (https://www.r-project.org/). Kaplan-Meier methodology was used to estimate survival curves and median survival times for time-to-event outcomes, and the log-rank test was used for statistical inference. Multivariable Cox proportional hazard regression models on CTCs were used to estimate the HR and its associated 95% CIs for time-to-event outcomes. These models included terms for standard pretreatment prognostic factors, including log_10_-transformed PSA, LDH, ALP, hemoglobin, NLR, ECOG performance score (ECOG PS) at baseline, androgen deprivation therapy (ADT) duration, age at screening, Gleason score, and presence of visceral metastasis at baseline (46). Treatment arm was also incorporated into the models to account for the fact that CTC count at baseline was not a stratification factor for randomization in CARD. A logistic regression model was used to estimate odds of tumor response according to baseline CTC and CTC-CIN counts, adjusting for the same variables as the Cox regression models. Parametric AFT Weibull models were fitted to estimate time-to-event outcomes depending on log-transformed baseline CTC and CTC-CIN counts. Fisher’s exact test was used to evaluate associations between groups defined by baseline CTC and CTC-CIN counts and resistance to treatment, defined radiologically as a BOR of progressive disease in RECIST 1.1–evaluable patients and biochemically as the lack of a PSA_50_ response. A *P* value less than 0.05 was considered significant.

### Study approval.

The CARD trial was approved by the institutional review board at each site as described in the Supplement and was conducted in compliance with the principles of the Declaration of Helsinki and Good Clinical Practice guidelines. Written informed consent was obtained from all participants. The study is registered with ClinicalTrials.gov (NCT02485691).

### Data availability.

Individual participant data will not be made publicly available due to privacy concerns. Data values for all figures are provided in the Supporting data value file. CARD is registered in Clinicaltrials.gov (NCT02485691), and the full study protocol and statistical analysis plan can be accessed therein (https://clinicaltrials.gov/study/NCT02485691).

## Author contributions

OL and JR planned the statistical analyses with input from JDB. JDB, SG, and RW led the CTC analyses. JR performed the statistical analyses. OL wrote the first draft of the manuscript with input from JR, NB, AS, SG, SM, CGR, BT, and OS. KP, EE, CS, DC, KF, BT, OS, SM, CGR, RDW, and JDB planned and conducted the CARD trial. OL, JR, NB, SG, and JDB had full access to CTC counts and clinical data. JDB had full access to all the data in the study and takes responsibility for the integrity of the data and the accuracy of the data analysis. Epic Sciences and Sanofi staff and all the coauthors reviewed and approved the manuscript. The authorship order for the co–first authors was established by alphabetical order of surnames.

## Funding support

- Sanofi sponsored and funded the clinical trial.

## Supplementary Material

Supplemental data

ICMJE disclosure forms

Supporting data values

## Figures and Tables

**Table 1:**
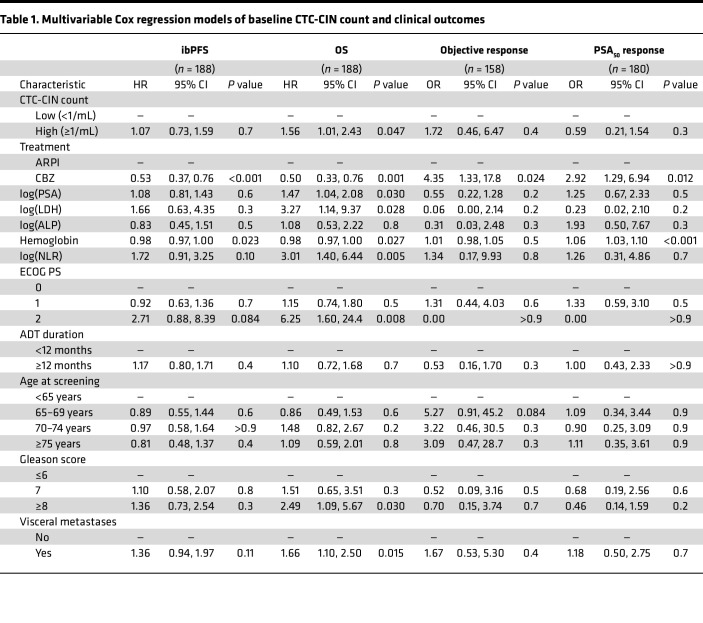
Multivariable Cox regression models of baseline CTC-CIN count and clinical outcomes

## Version 1 11/04/2025

In-Press Preview

## Version 2 12/22/2025

Electronic publication
