# Supplementary material for: Chromosomal instability in circulating tumor cells and cabazitaxel resistance in metastatic castration-resistant prostate cancer
Source: JCI Insight. 2025 Nov 4;10(24):e196505. doi: 10.1172/jci.insight.196505 (PMC12890519; doi:10.1172/jci.insight.196505)
Supplement: Supplemental data [file jciinsight-10-196505-s160.pdf]

Conflict of interest:

The CARD trial was funded by Sanofi. OL reports receiving travel support from Consens/Digital Science Press LLC. SG and RW were employees of Epic Sciences at the time this work was done.

KP reports receiving consulting fees from Sanofi. EE reports receiving consulting fees and honoraria from Johnson & Johnson, Pfizer, Astellas, and Bayer.

CS reports receiving consulting fees from Astellas Pharma, AstraZeneca, Bayer, Bristol Myers Squibb/Medarex, Foundation Medicine, Genzyme, Gilead, Merck, MSD, Pfizer, Janssen, Roche, Medscape, UroToday, and Tolmar and honoraria from Merck and Pfizer.

KF reports participating in advisory boards for Amgen, Astellas, AstraZeneca, Bayer, Clovis, Daiichi-Sankyo, Janssen, MSD, Novartis/AAA, Pfizer, Sanofi, Arvinas, CureVac, Macrogenics, and Orion Pharma. CGR and SM are Sanofi employees.

BT's institution has received grants from Bayer and Ferring, and he has received honoraria and consulting fees from Amgen, Astellas, AstraZeneca, Bayer, Ferring, Accord, MSD, Janssen, and Novartis.

AS has been the CI/PI of industry-sponsored clinical trials; reports receiving consulting fees from De Shaw Research, CHARM Therapeutics, Ellipses Pharma, and Droia Ventures; honoraria from Merck, Sharp and Dohme, and Astellas; and travel support from Nurix, Sanofi, and Roche-Genentech.

AS is named as coinventor for patent WO-2022129935-A1, which covers the use of a JMJD6 targeting agent for prostate cancer and participates in an independent safety data monitoring board for the ProBio Trial.

OS reports receiving grants from Amgen, AstraZeneca, Bayer, Johnson & Johnson, and Novartis; consulting fees from Abdera, Actithera, AdvanCell, Alpha9, Amgen, ART BioScience, Astellas Pharma, AstraZeneca, Bayer, Bavarian Nordic, Blue Earth Diagnostics, Bristol Myers Squibb, Clarity Pharmaceuticals, Convergent, Curium, Curdah, EMD Serono, Dendreon Isotopen Technologien, ITM Oncologics, Johnson & Johnson, Lantheus, Macrogenics, Merck, Modex, Myovant Sciences, Norroy, North Star, Novartis, Nucleus Biopharma, Noxopharm, Precede, Progenics, RATIO, Swiss Rockets, Telix Pharmaceuticals, Teneobio, and Wren Laboratories; and honoraria and travel support from Lantheus, NorthStar and Novartis.

OS participates on advisory boards for AstraZeneca, Merck and Pfizer; owns stock in AbbVie, Cardinal Health, Clarity Pharmaceuticals, Curadh, Lilly, Pfizer, Ratio, Telix, and United Health Group; and holds stock options with AdvanCell, Abdera, Actithera, ArtBio, Convergent, and Asta.

RDW reports consulting fees from Sanofi, Astellas, and Merck; honoraria from Astellas and Sanofi; and travel support from Sanofi and Bayer and participates in advisory boards for Merck and Astellas.

JDB has served on advisory boards and received fees from many companies, including Abbvie, Acai Therapeutics, Amgen, Amunix, Astellas, Bayer, Bioxcel Therapeutics, Celcuity, Crescendo, Daiichi, Dark Blue Therapeutics, Duke Street Bio Limited, Dunad Therapeutics, Endeavor Biomedicines, Genentech/Roche, GSK, Macrogenics, Merck Serono, MetaCurUm, Moma, Myricx, Novartis, Nurix Therapeutics, Nuvation Bio,

One-Carbon Therapeutics Inc., Oncternal, Orion, Page Therapeutics, Pfizer, Takeda, Tango Therapeutics, and Tubulis GmbH. He is an employee of the Institute of Cancer Research, which has received funding or other support for his research work from AstraZeneca, Cellcentric, Crescendo, Daiichi, Immunic Therapeutics, MetaCurUm, Myricx, Nurix Therapeutics, Oncternal, Orion, and Sanofi Aventis. The Institute of Cancer Research has a commercial interest in abiraterone, PARP inhibition in DNA repair–defective cancers, and PI3K/AKT pathway inhibitors.

JDB was named as an inventor, with no financial interest, for patent 8,822,438, submitted by Janssen, which covers the use of abiraterone acetate with corticosteroids. He has been the CI/PI of many industry-sponsored clinical trials.

JDB is a National Institute for Health Research (NIHR) Senior Investigator. The views expressed in this article are those of the author(s) and not necessarily those of the NHS, the NIHR, or the Department of Health.

Supplemental material

Supplemental Figure 1. Sample disposition.

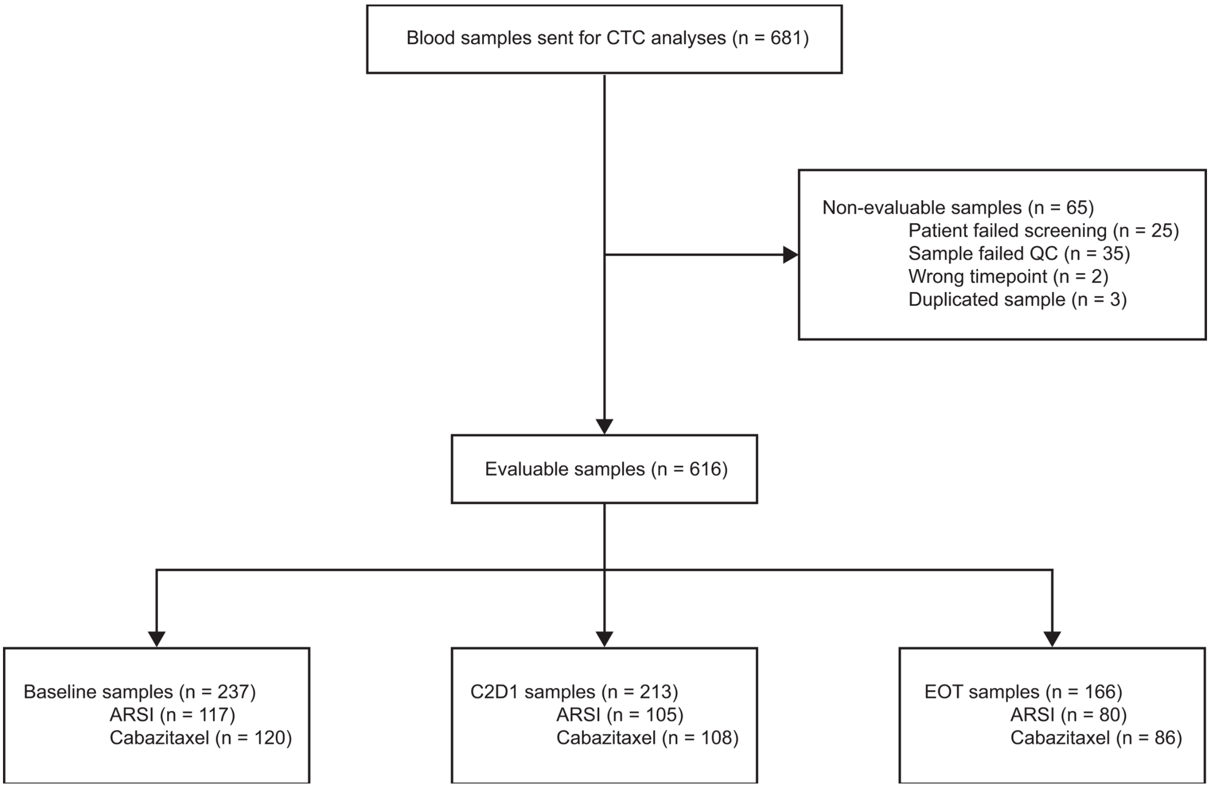

Supplemental Figure 2. Distribution of CTC (left) and CTC-CIN (right) counts at baseline (red), cycle 2 day 1 (green), and end of treatment (blue). Grey dots represent patients randomized to ARPI and red dots represent patients randomized to cabazitaxel. n = patients with minimum CTC or CTC-CIN count, N = total patients with evaluable samples per timepoint.

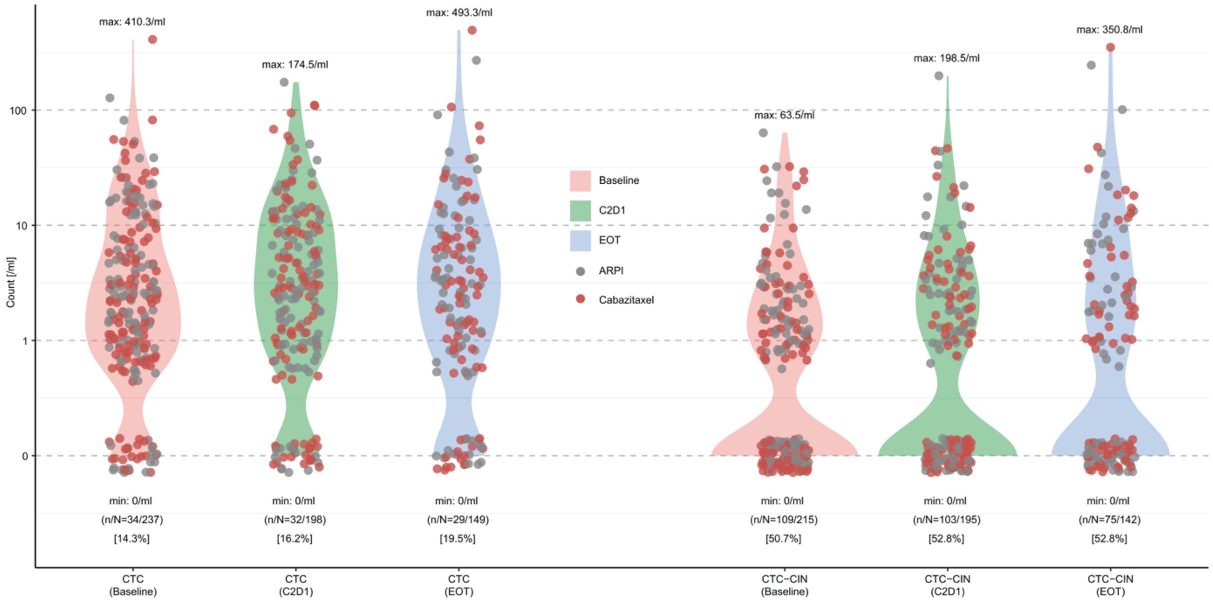

Supplemental Figure 3. Parametric accelerated failure time (AFT) Weibull models of OS, ibPFS, and time to PSA progression by baseline CTC-CIN count irrespective of treatment arm (left column) and adjusting for treatment arm (right column).

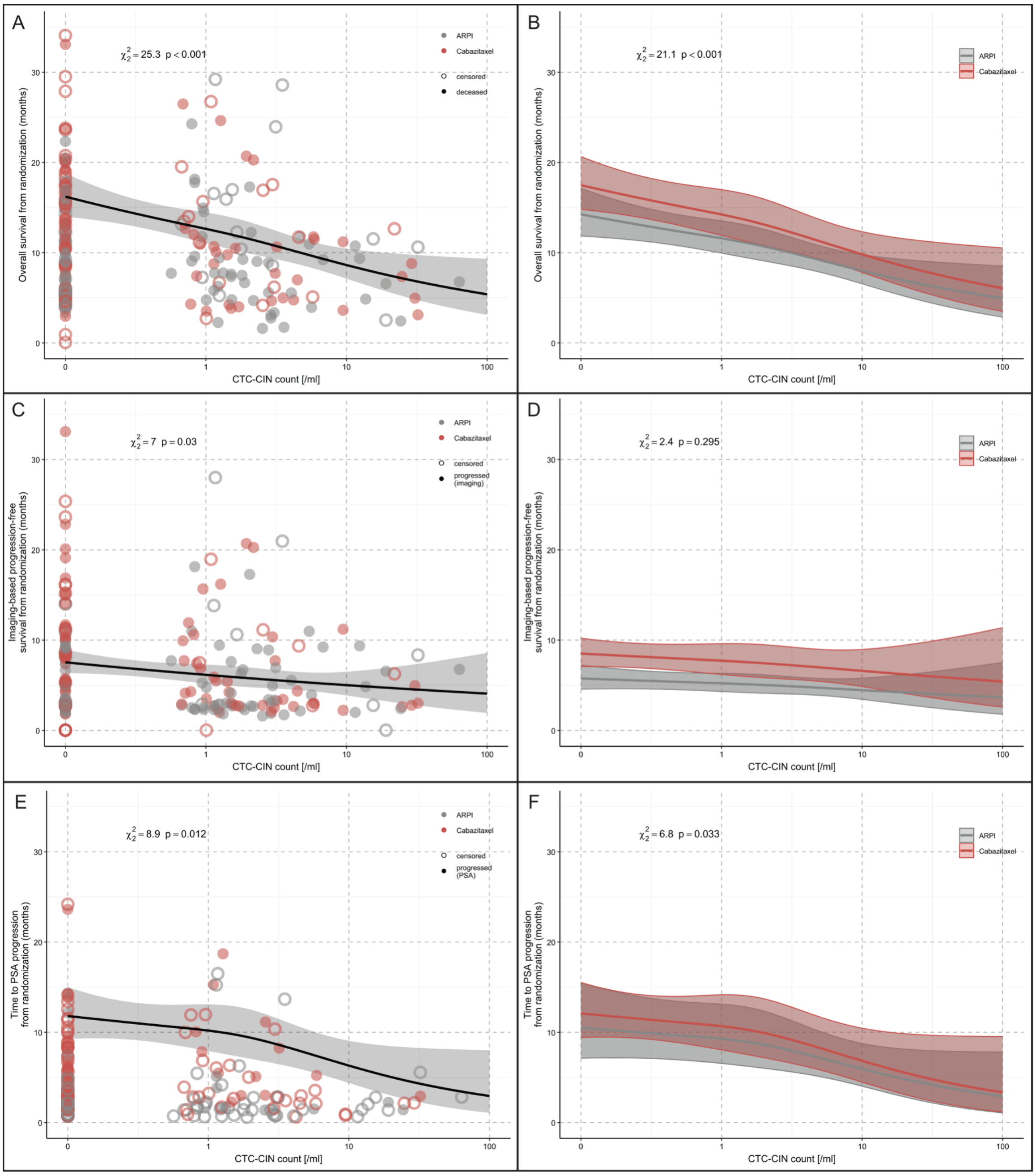

Supplemental Figure 4. Parametric accelerated failure time (AFT) Weibull models of OS, ibPFS, and time to PSA progression by baseline CTC count irrespective of treatment arm (left column) and adjusting for treatment arm (right column).

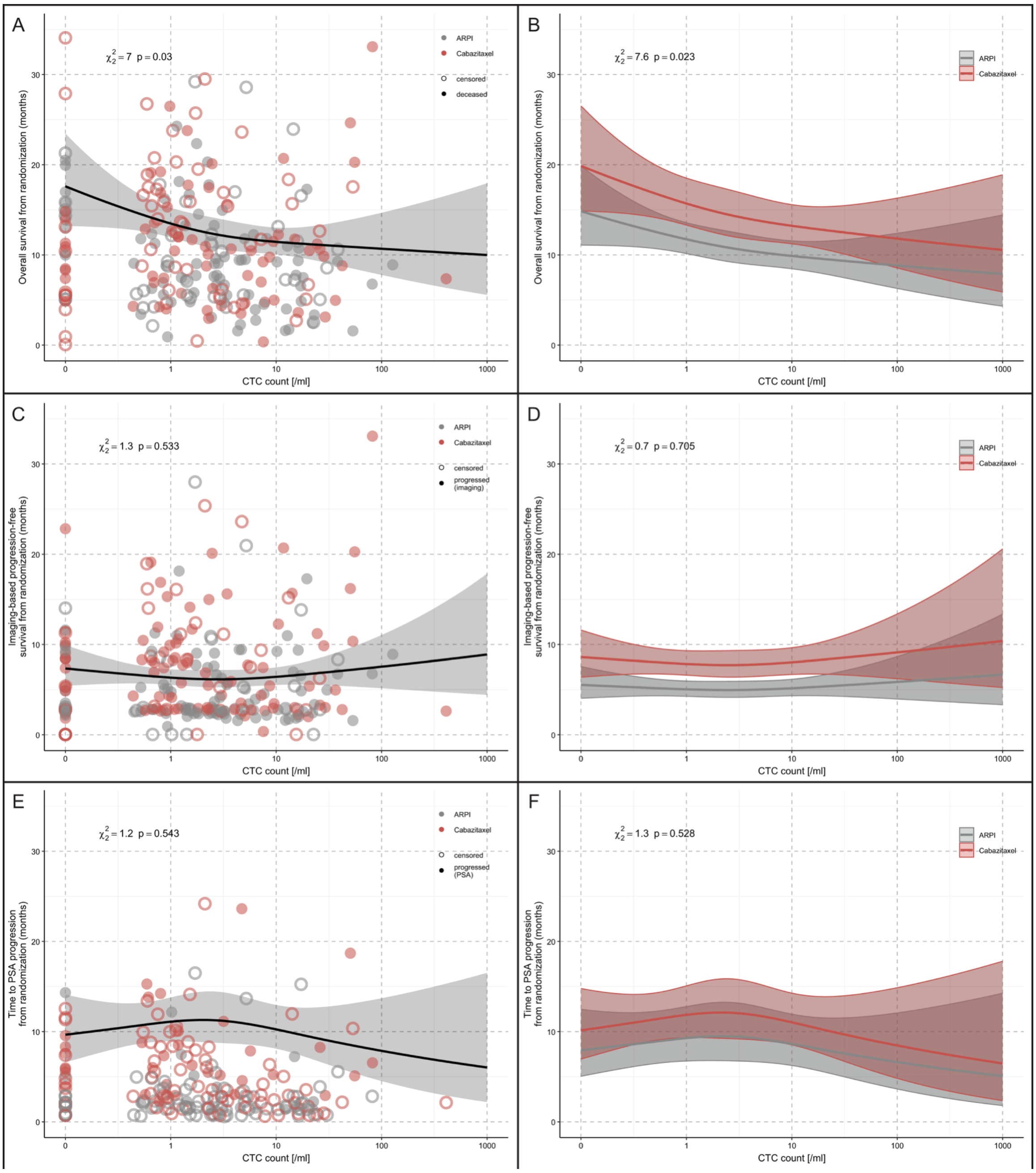

Supplemental Figure 5. Swimmer plot of duration of RECIST 1.1 response by CTC-CIN counts at baseline and treatment arm. Continuous line marks 24 weeks after randomization.

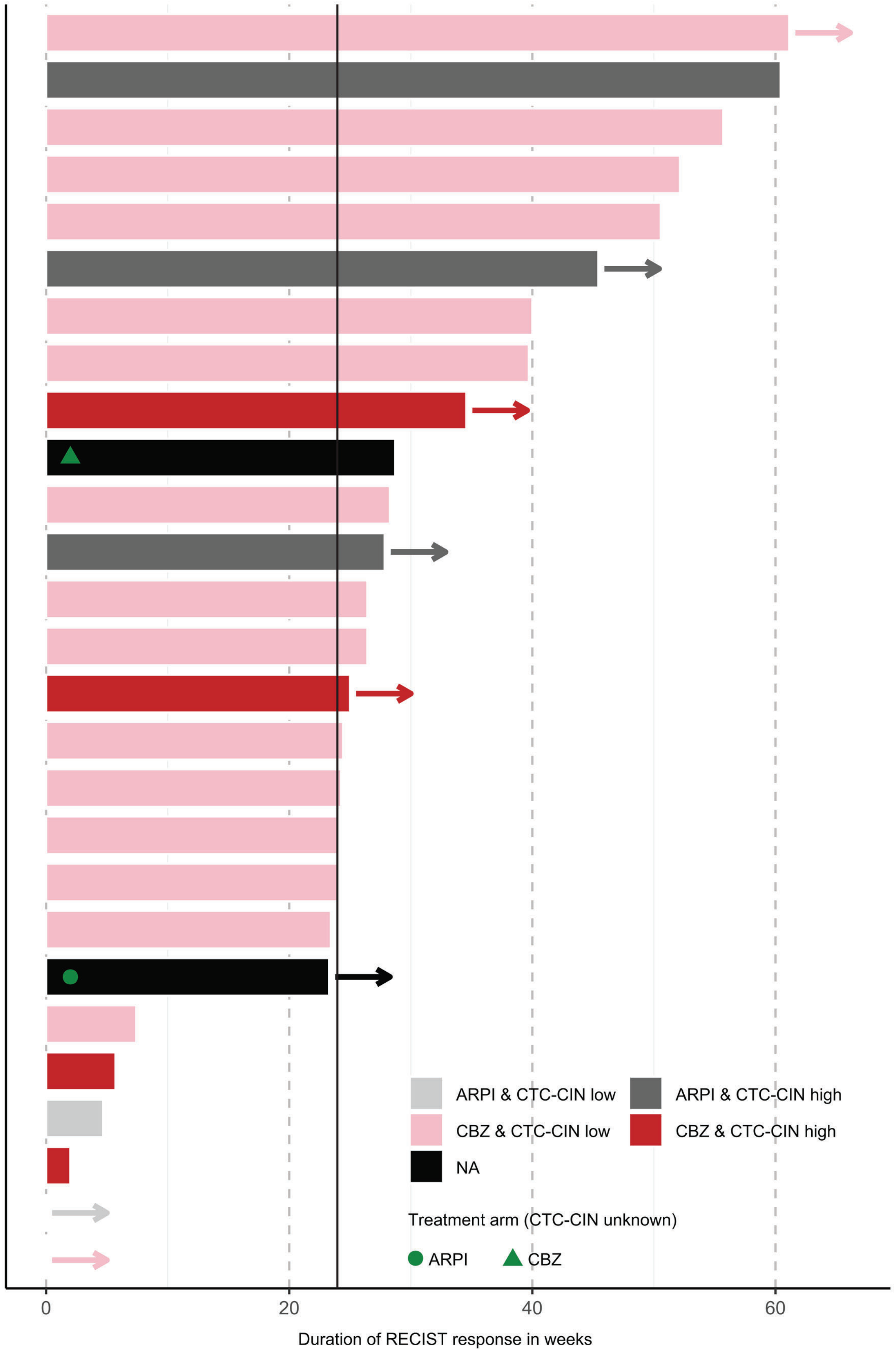

Supplemental Figure 6. Swimmer plot of duration of PSA response by CTC-CIN count at baseline and treatment arm. Continuous line marks 24 weeks after randomization.

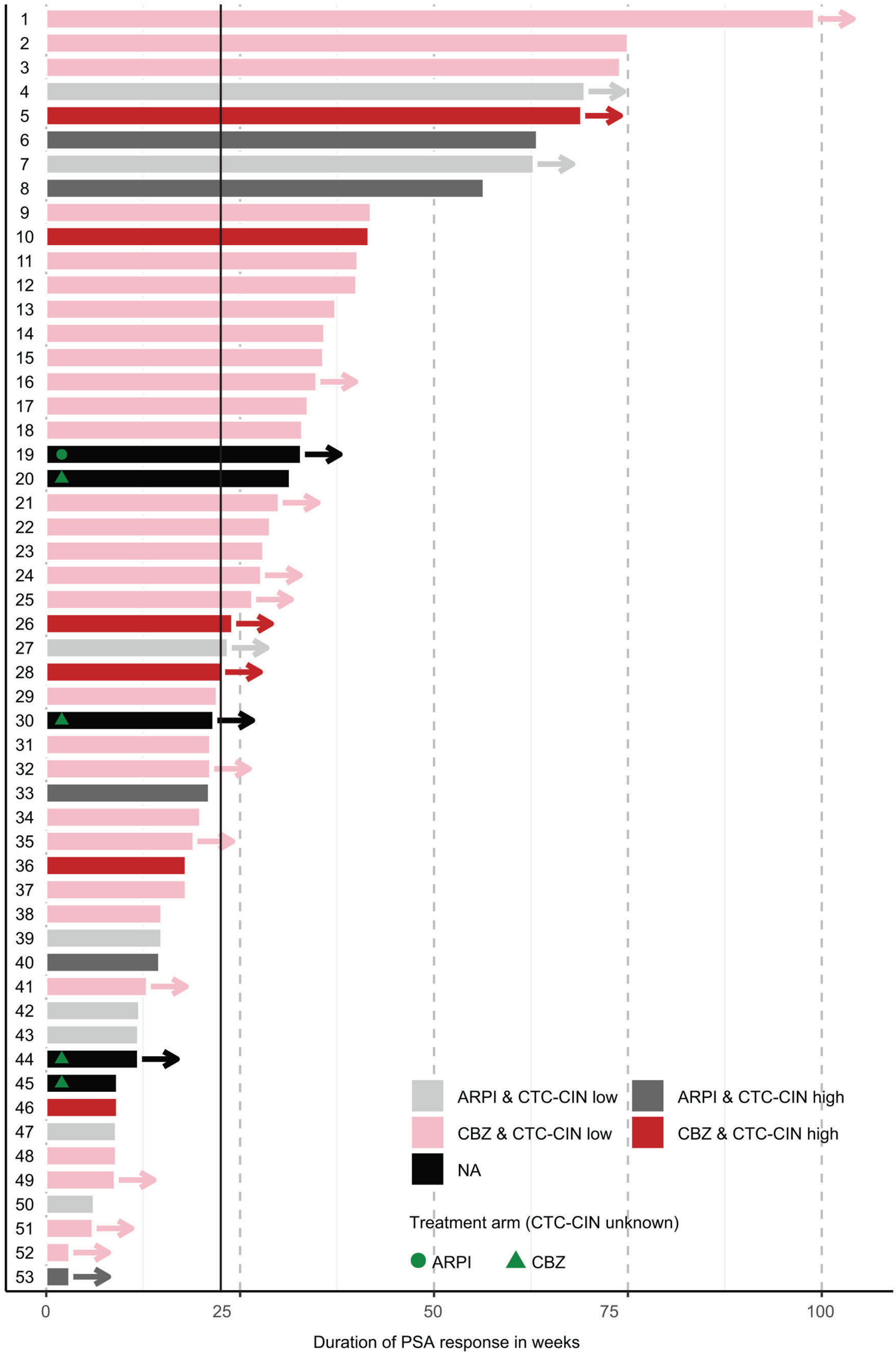

Supplemental Figure 7. Kaplan-Meier curves of OS from randomization by baseline CTC and CTC-CIN counts (cut-off for CTC, 2/mL; cut-off for CTC-CIN, 1/mL).

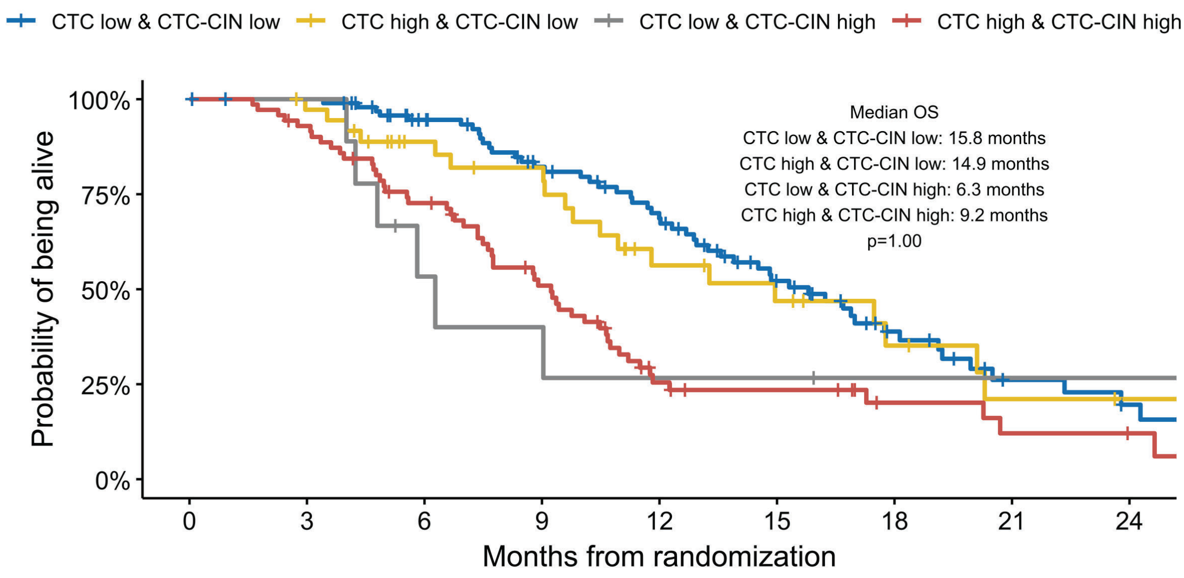

Number at risk (number of events)

	0	3	6	9	12	15	18	21	24
CTC low & CTC-CIN low	98 (0)	96 (0)	80 (5)	63 (15)	50 (25)	31 (36)	17 (43)	8 (48)	5 (50)
CTC high & CTC-CIN low	37 (0)	35 (1)	26 (4)	23 (6)	13 (13)	10 (15)	6 (17)	3 (19)	2 (19)
CTC low & CTC-CIN high	9 (0)	9 (0)	4 (4)	3 (5)	2 (6)	2 (6)	1 (6)	1 (6)	1 (6)
CTC high & CTC-CIN high	71 (0)	65 (5)	49 (19)	32 (33)	13 (48)	10 (49)	5 (50)	3 (52)	2 (52)

Supplemental Table 1. Data availability for CTC and CTC-CIN counts by timepoint and treatment arm.

	Baseline	C2D1	EOT
**Both arms**			
CTC	237	198	149
CTC-CIN	215	195	142
**ARPI only**			
CTC	117	98	72
CTC-CIN	106	96	68
**Cabazitaxel only**			
CTC	120	100	77
CTC-CIN	109	99	74

Supplemental Table 2. CTC and CTC-CIN counts by timepoint and treatment arm.				
**Characteristic**	**N**	**ARPI**, N = 117	**Cabazitaxel**, N = 120	**Overall**, N = 237
**CTC (Baseline)**	237			
Median (Q1, Q3)		2.47 (0.80, 7.56)	1.39 (0.57, 6.05)	2.03 (0.63, 7.09)
Min, Max		0.00, 127.48	0.00, 410.17	0.00, 410.17
**CTC (C2D1)**	198			
Median (Q1, Q3)		2.57 (0.83, 8.41)	2.76 (0.58, 9.31)	2.70 (0.76, 8.98)
Min, Max		0.00, 174.40	0.00, 110.41	0.00, 174.40
**CTC (EOT)**	149			
Median (Q1, Q3)		2.52 (0.52, 7.66)	2.34 (0.58, 7.14)	2.38 (0.55, 7.50)
Min, Max		0.00, 270.16	0.00, 493.20	0.00, 493.20
**CTC-CIN (baseline)**	215			
Median (Q1, Q3)		0.73 (0.00, 1.99)	0.00 (0.00, 1.32)	0.00 (0.00, 1.69)
Min, Max		0.00, 63.43	0.00, 32.20	0.00, 63.43
**CTC-CIN (C2D1)**	195			
Median (Q1, Q3)		0.68 (0.00, 2.53)	0.00 (0.00, 2.27)	0.00 (0.00, 2.43)
Min, Max		0.00, 198.40	0.00, 46.37	0.00, 198.40
**CTC-CIN (EOT)**	142			
Median (Q1, Q3)		0.00 (0.00, 2.95)	0.37 (0.00, 2.31)	0.00 (0.00, 2.62)
Min, Max		0.00, 245.23	0.00, 350.72	0.00, 350.72

Supplemental Table 3. Baseline characteristics by treatment arm and baseline CTC-CIN count.

	ARPI				Cabazitaxel			
**Characteristic**	**N**	**Overall**<br>N = 106[1]	**CTC-CIN low**<br>(< 1/ml)<br>N = 59[1]	**CTC-CIN high**<br>(≥ 1/ml)<br>N = 47[1]	**N**	**Overall**<br>N = 109[1]	**CTC-CIN low**<br>**(< 1/ml)**<br>N = 76[1]	**CTC-CIN high**<br>**(≥ 1/ml)**<br>N = 33[1]
**Age at screening**	106				109			
< 65 years		28 (26%)	17 (29%)	11 (23%)		26 (24%)	17 (22%)	9 (27%)
65 - 69 years		22 (21%)	12 (20%)	10 (21%)		28 (26%)	17 (22%)	11 (33%)
70 - 74 years		27 (25%)	17 (29%)	10 (21%)		19 (17%)	16 (21%)	3 (9.1%)
≥ 75 years		29 (27%)	13 (22%)	16 (34%)		36 (33%)	26 (34%)	10 (30%)
**ECOG PS**	106				109			
0		49 (46%)	32 (54%)	17 (36%)		47 (43%)	36 (47%)	11 (33%)
1		54 (51%)	26 (44%)	28 (60%)		59 (54%)	39 (51%)	20 (61%)
2		3 (2.8%)	1 (1.7%)	2 (4.3%)		3 (2.8%)	1 (1.3%)	2 (6.1%)
**ADT duration**	105				108			
< 12 months		48 (46%)	25 (43%)	23 (49%)		51 (47%)	35 (47%)	16 (48%)
≥ 12 months		57 (54%)	33 (57%)	24 (51%)		57 (53%)	40 (53%)	17 (52%)
**Gleason score**	103				101			
≤ 6		9 (8.7%)	4 (6.8%)	5 (11%)		15 (15%)	10 (14%)	5 (16%)
7		25 (24%)	11 (19%)	14 (32%)		26 (26%)	15 (22%)	11 (34%)
≥ 8		69 (67%)	44 (75%)	25 (57%)		60 (59%)	44 (64%)	16 (50%)
**Pain**	98	76 (78%)	36 (69%)	40 (87%)	103	72 (70%)	49 (68%)	23 (74%)
**Visceral metastases**	106	37 (35%)	19 (32%)	18 (38%)	109	41 (38%)	24 (32%)	17 (52%)
**PSA [ng/mL]**	103	63 (19, 223)	39 (16, 183)	120 (26, 249)	105	61 (12, 162)	39 (11, 132)	104 (44, 283)
**LDH [IU/L]**	103	251 (199, 369)	224 (188, 322)	311 (236, 407)	107	243 (202, 353)	223 (193, 334)	305 (239, 439)
**ALP [IU/L]**	105	124 (77, 223)	97 (74, 144)	173 (108, 549)	107	129 (86, 237)	114 (75, 194)	193 (107, 336)
**Hemoglobin [g/dL]**	106	123 (110, 134)	124 (114, 136)	121 (109, 133)	107	121 (112, 132)	126 (115, 135)	117 (110, 121)
**NLR [unitless]**	103	3.40 (2.35, 5.03)	3.40 (2.21, 4.69)	3.44 (2.35, 6.00)	106	3.4 (2.3, 6.1)	3.1 (2.1, 5.6)	3.8 (3.0, 8.6)

[1]n (%); Median (IQR)

Supplemental Table 4. Baseline characteristics by treatment arm and baseline CTC count.

		ARPI				Cabazitaxel		
**Characteristic**	**N**	**Overall** N = 117[1)]	**CTC low** (< 2/ml) N = 52[1]	**CTC high** (≥ 2/ml) N = 65[1]	**N**	**Overall** N = 120[1]	**CTC low** (< 2/ml) N = 65[1]	**CTC high** (≥ 2/ml) N = 55[1]
**Age at screening**	117				120			
< 65 years		30 (26%)	21 (40%)	9 (14%)		27 (23%)	17 (26%)	10 (18%)
65 - 69 years		24 (21%)	8 (15%)	16 (25%)		30 (25%)	13 (20%)	17 (31%)
70 - 74 years		31 (26%)	12 (23%)	19 (29%)		21 (18%)	10 (15%)	11 (20%)
≥ 75 years		32 (27%)	11 (21%)	21 (32%)		42 (35%)	25 (38%)	17 (31%)
**ECOG PS**	117				120			
0		53 (45%)	29 (56%)	24 (37%)		51 (43%)	31 (48%)	20 (36%)
1		61 (52%)	21 (40%)	40 (62%)		66 (55%)	33 (51%)	33 (60%)
2		3 (2.6%)	2 (3.8%)	1 (1.5%)		3 (2.5%)	1 (1.5%)	2 (3.6%)
**ADT duration**	116				119			
< 12 months		54 (47%)	25 (48%)	29 (45%)		54 (45%)	27 (42%)	27 (49%)
≥ 12 months		62 (53%)	27 (52%)	35 (55%)		65 (55%)	37 (58%)	28 (51%)
**Gleason score**	112				112			
≤ 6		9 (8.0%)	3 (5.9%)	6 (9.8%)		17 (15%)	10 (16%)	7 (14%)
7		27 (24%)	6 (12%)	21 (34%)		28 (25%)	11 (18%)	17 (33%)
≥ 8		76 (68%)	42 (82%)	34 (56%)		67 (60%)	40 (66%)	27 (53%)
**Pain**	108	83 (77%)	35 (73%)	48 (80%)	113	81 (72%)	42 (67%)	39 (78%)
**Visceral metastases**	117	40 (34%)	16 (31%)	24 (37%)	120	43 (36%)	24 (37%)	19 (35%)
**PSA [ng/mL]**	114	62 (19, 235)	36 (12, 198)	104 (26, 238)	116	61 (14, 160)	36 (9, 118)	95 (30, 243)
**LDH [IU/L]**	112	258 (199, 390)	239 (195, 335)	302 (208, 403)	117	247 (202, 351)	219 (193, 337)	266 (214, 397)
**ALP [IU/L]**	114	126 (77, 233)	98 (72, 161)	141 (95, 399)	117	126 (81, 235)	105 (76, 169)	176 (95, 288)
**Hemoglobin [g/dL]**	115	122 (110, 134)	124 (111, 136)	120 (110, 130)	117	121 (113, 131)	125 (112, 135)	119 (113, 129)
**NLR [unitless]**	112	3.39 (2.35, 4.99)	3.24 (1.95, 4.56)	3.49 (2.57, 5.54)	116	3.4 (2.3, 5.8)	3.0 (1.9, 5.3)	3.8 (2.8, 7.4)

[1]n (%); Median (IQR)

Supplemental Table 5. Multivariable Cox regression models of baseline CTC count and clinical outcomes.

	ibPFS (n = 205)			OS (n = 205)			Objective response (n = 168)			PSA_{50} response (n = 190)		
**Characteristic**	**HR[1]**	**95% CI[1]**	**p-value**	**HR[1]**	**95% CI[1]**	**p-value**	**OR[1]**	**95% CI[1]**	**p-value**	**OR[1]**	**95% CI[1]**	**p-value**
**CTC count**												
Low (< 2/ml)	—	—		—	—		—	—		—	—	
High (≥ 2/ml)	1.14	0.80, 1.63	0.5	1.43	0.95, 2.13	0.083	0.86	0.26, 2.81	0.8	0.56	0.23, 1.34	0.2
**Treatment**												
ARPI	—	—		—	—		—	—		—	—	
CBZ	0.50	0.36, 0.70	**<0.001**	0.53	0.36, 0.77	**0.001**	3.85	1.23, 14.9	**0.030**	3.21	1.49, 7.30	**0.004**
**log(PSA)**	1.12	0.85, 1.48	0.4	1.68	1.21, 2.33	**0.002**	0.52	0.21, 1.20	0.14	1.26	0.69, 2.30	0.4
**log(LDH)**	1.70	0.71, 4.10	0.2	2.64	1.02, 6.83	**0.045**	0.16	0.00, 4.20	0.3	0.62	0.07, 4.90	0.7
**log(ALP)**	0.90	0.53, 1.54	0.7	1.25	0.68, 2.32	0.5	0.37	0.04, 2.82	0.4	1.38	0.38, 4.92	0.6
**Hemoglobin**	0.99	0.97, 1.00	**0.020**	0.98	0.97, 1.00	**0.035**	1.01	0.97, 1.05	0.7	1.06	1.02, 1.09	**0.001**
**log(NLR)**	1.58	0.84, 2.98	0.2	2.50	1.21, 5.19	**0.014**	1.26	0.16, 8.75	0.8	1.21	0.32, 4.40	0.8
**ECOG PS**												
0	—	—		—	—		—	—		—	—	
1	1.01	0.70, 1.45	>0.9	1.17	0.78, 1.76	0.5	1.43	0.50, 4.23	0.5	1.23	0.56, 2.75	0.6
2	2.78	0.92, 8.36	0.069	4.47	1.21, 16.5	**0.024**	0.00		>0.9	0.00		>0.9
**ADT duration**												
< 12 months	—	—		—	—		—	—		—	—	
≥ 12 months	1.17	0.81, 1.68	0.4	1.09	0.73, 1.64	0.7	0.50	0.15, 1.53	0.2	0.88	0.40, 1.96	0.8
**Age at screening**												
< 65 years	—	—		—	—		—	—		—	—	
65 - 69 years	0.93	0.58, 1.51	0.8	0.96	0.55, 1.66	0.9	4.99	0.89, 41.7	0.090	1.20	0.39, 3.68	0.8
70 - 74 years	0.94	0.56, 1.57	0.8	1.48	0.84, 2.63	0.2	3.03	0.46, 27.1	0.3	0.94	0.27, 3.17	>0.9
≥ 75 years	0.79	0.48, 1.30	0.4	1.13	0.64, 1.97	0.7	3.28	0.55, 28.0	0.2	1.33	0.44, 4.07	0.6
**Gleason score**												
≤ 6	—	—		—	—		—	—		—	—	
7	1.08	0.59, 1.97	0.8	1.26	0.59, 2.67	0.6	0.60	0.11, 3.39	0.5	0.78	0.22, 2.85	0.7
≥ 8	1.33	0.74, 2.39	0.3	1.84	0.89, 3.82	0.10	0.75	0.17, 3.66	0.7	0.59	0.19, 1.93	0.4
**Visceral metastases**												
No	—	—		—	—		—	—		—	—	
Yes	1.38	0.96, 1.97	0.079	1.57	1.07, 2.31	**0.022**	1.72	0.58, 5.19	0.3	0.91	0.40, 2.01	0.8

1HR = Hazard Ratio, CI = Confidence Interval, OR = Odds Ratio

Supplemental Table 6. Best radiological response by treatment arm and baseline CTC and CTC-CIN count.

		ARPI			CBZ		
**Group**	**Parameter**	**N**	**Low[1]**	**High[1]**	**N**	**Low[1]**	**High[1]**
Baseline CTC count		86			104		
	CR[1]		0 (0%)	0 (0%)		0 (0%)	0 (0%)
	PR[1]		1 (2%)	5 (11%)		15 (26%)	6 (13%)
	SD[1]		23 (58%)	25 (54%)		33 (57%)	24 (52%)
	PD[1]		16 (40%)	16 (35%)		10 (17%)	16 (35%)
Baseline CTC-CIN count		81			97		
	CR[1]		0 (0%)	0 (0%)		0 (0%)	0 (0%)
	PR[1]		2 (4%)	3 (9%)		16 (23%)	4 (14%)
	SD[1]		26 (54%)	19 (58%)		42 (61%)	9 (32%)
	PD[1]		20 (42%)	11 (33%)		11 (16%)	15 (54%)

[1]n (%); CR = Complete Response; PR = Partial Response; SD = Stable Disease; PD = Progressive Disease

Supplemental Table 7. Contingency tables for progressive vs non progressive disease as best overall response by treatment arm and baseline CTC and CTC-CIN count.

	ARPI					CBZ				
**Group**	**PD**[1]	**Non-PD**[1]	**OR**[1]	**95% CI**[1]	**p-value**	**PD**[1]	**Non-PD**[1]	**OR**[1]	**95% CI**[1]	**p-value**
CTC low	16 (40%)	24 (60%)	1.25	0.47, 3.29	0.66	10 (17%)	48 (83%)	0.39	0.14, 1.06	0.07
CTC high	16 (35%)	30 (65%)				16 (35%)	30 (65%)			
CTC-CIN low	20 (42%)	28 (58%)	1.42	0.52, 4.03	0.49	11 (16%)	58 (84%)	0.17	0.05, 0.49	**<0.001**
CTC-CIN high	11 (33%)	22 (67%)				15 (54%)	13 (46%)			

[1]n (%); PD = Progressive Disease; OR = Odds Ratio; CI = Confidence Interval

Supplemental Table 8. PSA_{50} response rate by treatment arm and baseline CTC and CTC-CIN count.

Group	Parameter	ARPI (PSA_{50} RR, 95% CI)[12]	CBZ (PSA_{50} RR, 95% CI)[12]	Overall (PSA_{50} RR, 95% CI)[2]
Baseline CTC count	Low	8/47; 17% (7.6%, 31%)	24/57; 42% (29%, 56%)	32/104; 31% (22%, 41%)
	High	6/55; 11% (4.1%, 22%)	15/50; 30% (18%, 45%)	21/105; 20% (13%, 29%)
Baseline CTC-CIN count	Low	8/57; 14% (6.3%, 26%)	29/70; 41% (30%, 54%)	37/127; 29% (21%, 38%)
	High	5/40; 13% (4.2%, 27%)	6/30; 20% (7.7%, 39%)	11/70; 16% (8.1%, 26%)

[1]n/No. obs.; %; [2]CI = Confidence Interval

Supplemental Table 9. Contingency tables for absence vs presence of a PSA_{50} reponse by treatment arm and baseliine CTC and CTC-CIN count group.

	ARPI					CBZ				
**Group**	**Non-PSA_{50} response[1]**	**PSA_{50} response[1]**	**OR[1]**	**95% CI[1]**	**p-value**	**Non-PSA_{50} response[1]**	**PSA_{50} response[1]**	**OR[1]**	**95% CI[1]**	**p-value**
CTC low	39 (83%)	8 (17%)	0.16, 2.16	0.47, 3.29	0.4	33 (58%)	24 (42%)	0.39	0.59	0.23
CTC high	49 (89%)	6 (11%)				35 (70%)	15 (30%)			
CTC-CIN low	49 (86%)	8 (14%)	0.21, 3.34	0.52, 4.03	1	41 (59%)	29 (41%)	0.17	0.36	**0.04**
CTC-CIN high	35 (87%)	5 (13%)				24 (80%)	6 (20%)			

[1]n (%); OR = Odds Ratio; CI = Confidence Interval

CARD trial sites

a. Austria
    i. Medical University of Vienna, Vienna, Austria
    ii. Ordensklinikum Linz GmbH Elisabethinen, Linz, Austria
b. Belgium
    i. Erasme Hospital, Brussels, Belgium
    ii. Ghent University Hospital, Ghent, Belgium
    iii. Institut Jules Bordet, Bruxelles, Belgium
    iv. Grand Hôpital de Charleroi, Charleroi, Belgium
    v. AZ Sint-Lucas, Brugge, Belgium
    vi. KU Leuven-University Hospital of Leuven, Leuven, Belgium
c. Czech Republic
    i. Palacky University Medical School and Teaching Hospital, Olomouc, Czech Republic
    ii. Medical School and University Hospital in Pilsen, Pilsen, Czech Republic
    iii. Masarykuv Onkologicky Ustav, Brno, Czech Republic
    iv. Thomayerova Nemocnice, Praha, Czech Republic
d. France
    i. Jean Godinot Institute, Reims, France
    ii. Foch Hospital, Suresnes, France

iii. Jean Perrin Center, Clermont Ferrand, France
iv. Strasbourg University Hospital, Strasbourg, France
v. Saint Louis Hospital, Paris, France
vi. J Paolii Calmettes Institute, Marseille, France
vii. Institut Gustave Roussy and University of Paris Sud, Villejuif, France
viii. CHU Bretonneau, and University François Rabelais, Tours, France
ix. Centre Val D Aurelle, Montpellier, France
x. Centre Léon Bérard, Lyon, France
xi. ARIO (Centre armoricain de radiologie, imagerie médicale et oncologie), Plerin, France

e. Germany
   i. Studienpraxis Urologie, Nürtingen, Germany
   ii. Urologicum Duisburg, Duisburg, Germany
   iii. University Medical Centre Mannheim, Mannheim, Germany
   iv. Universitaetsklinikum Muenster, Muenster, Germany
   v. Onkologie Aschaffenburg, Aschaffenburg, Germany
   vi. Jena University Hospital, Jena
   vii. Wissenschaftskontor Nord GmbH & Co. KG, Rostock, Germany
   viii. Kliniken Essen-Mitte Evang. Huyssens-Stiftung/Knappschaft GmbH, Germany
   ix. Aturo-Urologische, Gemeinschaftspraxis, Berlin, Germany

x. University Hospital Schleswig-Holstein, Campus Lübeck, Lübeck, Germany
xi. University of Magdeburg, Magdeburg, Germany
xii. University Medical Center, Göttingen, Germany.
xiii. Radboud University Nijmegen Medical Center, Nijmegen, The Netherlands.

f. Greece
i. Alexandra Hospital, National and Kapodistrian University of Athens, Athens, Greece
ii. Papageorgiou General Hospital of Thessaloniki, Thessaloniki, Greece
iii. Athens Medical Center, Marousi, Athens, Greece

g. Iceland
i. Landspitali University Hospital, Reykjavik, Iceland

h. Ireland
i. Mater Misericordiae University Hospital, Ireland
ii. Adelaide and Meath Hospital Tallaght, Dublin, Ireland

i. Italy
i. Azienda Ospedaliera Universitaria Integrata (AOUI), Verona & Policlinico Universitario Agostino Gemelli IRCCS, Rome, Italy
ii. Azienda Ospedaliera San Camillo Forlanini, Rome, Italy
iii. Istituto Nazionale Tumori–IRCCS-Fondazione G. Pascale, Naples, Italy
iv. Azienda Ospedaliero Universitaria Di Parma, Parma, Italy

    v. Institute for Cancer Research and Treatment of Candiolo, Candiolo, Italy
    vi. Brescia Civil Hospital, Brescia, Italy
    vii. University Hospital of Pisa, Pisa, Italy

j. Norway
    i. Østfold Hospital Trust, Grålum, Norway
    ii. Trondheim University Hospital, Trondheim, Norway

k. Spain
    i. Hospital Universitario 12 de Octubre, Madrid, Spain
    ii. Vall d'Hebron University Hospital, Barcelona, Spain
    iii. Hospital Universitario Virgen Del Rocio, Sevilla, Spain
    iv. Hospital la Paz, Castellana, Spain

l. The Netherlands
    i. Erasmus Medical Center, Rotterdam, The Netherlands
    ii. Zuyderland Medisch Centrum, Sittard-Geleen, The Netherlands
    iii. Amphia Hospital, Breda, The Netherlands

m. United Kingdom
    i. The Institute of Cancer Research and the Royal Marsden Hospital, London, United Kingdom
